# The first patient treated with a triple combination therapy after recurrent ischemic stroke

**DOI:** 10.25122/jml-2019-1006

**Published:** 2019

**Authors:** Andreas Winkler, Irmgard Zelenka, Elisabeth Schweng, Jan Skabrada, Ines Schandl, Andreas Janecek

**Affiliations:** Department of Neurological Rehabilitation, Pirawarth Clinic, Bad Pirawarth, Austria

## Abstract

This case report presents the evolution of a patient with recurrent ischemic stroke, in the context of treatment with multimodal agent cerebrolysin, as an add-on to neurological rehabilitation and tDCS therapy. The patient was evaluated before and after treatment using a battery of tests such as the Nine-Hole Peg Test, handgrip force, Functional Hand Scale (1-5), Action Research Arm Test (ARAT), Active Range of Motion (AROM) for the left shoulder, registering visible improvements in functional motor recovery after this therapeutic combination.

## Background

Stroke is not only an acute killer but remains the leading cause of long-term disability [1]. Big trials testing different drugs like Dopamine, SSRI or Amphetamine for promoting recovery after stroke have had disappointing results. However, the neuropeptide preparation Cerebrolysin® has shown to promote upper limb recovery when given early after stroke, but there are no available data for stroke patients in the more subacute or chronic phase after stroke [2]. Cerebrolysin® is thought to exert its restorative effects after stroke by enhancing the levels of neurotrophic factors in the brain and mimicking especially the effects of Brain Derived Neurotrophic Factor (BDNF) [3].

Brain-derived neurotrophic factor (BDNF) and its receptor tropomyosin-related kinase type B are actively produced in several regions of the brain (e.g., hippocampus, cortex, basal ganglia) and are involved in regulating neuronal activity. Further, BDNF has been shown to play a role in both the protection and recovery of functions after stroke. However, BDNF needs to reach a threshold level to have any effect on recovery. After a stroke, patients (especially those over 65 years old and those with severe stroke or in the chronic phase) show reduced plasma levels of BDNF [1]. Enhancing the brain levels of NTFs, the application of Cerebrolysin® has shown to promote recovery even in the chronic states after stroke in several preclinical experiments. Additionally, BDNF has been shown to be the primary mediator of the neuroplastic effects induced by anodal tDCS [4]. In particular, recovery of motor functions after stroke is mediated via the activity-dependent release of BDNF, induced either by atDCS or active motor training, with BDNF levels needing to reach a threshold to facilitate enhancement in neuronal plasticity and post-stroke recovery [4].

## Case Description

A 69-year-old male patient was admitted to our rehabilitation facility at the Pirawarth Clinic following a second, probably embolic stroke of undetermined source (ESUS) in the area of the right middle cerebral artery in March 2017 (proximal ACMD, M1 segment). He suffered a first ischemic stroke in the same vascular area on October 2016. When he was admitted to our clinic in May 2017, he already had completed four weeks of rehabilitation as an inpatient in another rehabilitation facility with only minor improvements in his upper limb motor functions.

**Figure 1:**
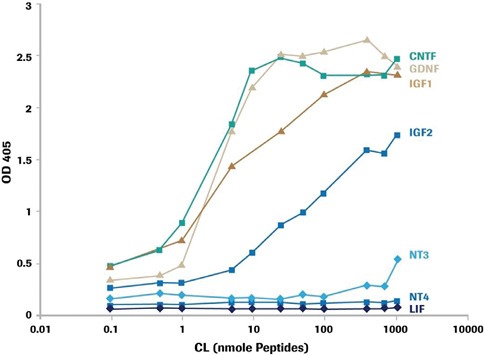
Cerebrolysin® enhances levels of Neurotrophins

The patient presented with left-sided hemiparesis, left hemianopia, and left hemineglect. He was able to walk with a cane and the hemiparesis was affecting especially his left upper limb with distal weakness (distal MCR Grade 1-2, proximal MCR Grade 3-4). He was not able to manipulate small parts or perform isolated finger movements (e.g., to pick up coins, use the cutlery and so forth).

The patient was treated with 75 mg of Clopidogrel in combination with Aspirin (100 mg) for secondary stroke prevention. His diabetes, hypertension, and hyperlipidemia were well-controlled medically. As he already was an ambulatory patient, the primary goal of our rehabilitation efforts was to improve the motor activity-level of his left upper extremity and to promote manual dexterity.

In early June 2017, we decided to offer the patient a two weeks course of an innovative multimodal therapy, including:

1. intensive occupational therapy with a minimum of one hour of task-oriented training per day during tDCS stimulation; additional conventional rehabilitation therapy for 2-3 hours per day for 28 days
2. daily anodal transcranial direct current stimulation (anodal tDCS 2x20 minutes, over the left motor cortex M1), five days a week from Monday to Friday.
3. A 2-week treatment course with daily intravenous infusions of 30 mL Cerebrolysin for 14 days.

## Intervention

Before and after providing 14 days of this triple combination therapy, we assessed specific outcomes of arm function and hand dexterity (Table 1). The patient’s ARAT (Action Research Arm Test) score improved from 38 to 49 out of 57 points (corresponding to a 60% proportional recovery rate). This presents a clinically meaningful improvement (>6 points) as the patient was now able to perform fine motor tasks with his paretic hand and was able to transfer these improvements into motor tasks of everyday use (picking up small pieces, performing a pinch grip and using the cutlery properly). He also improved manual speed and dexterity functions (Nine Hole Peg Test) and expanded his active range of motion of the left shoulder, especially arm abduction. He also slightly improved handgrip strength, but this was not clinically meaningful.

**Table 1:** Baseline and post-treatment evaluation on treatment day 14

Test	Beginning	End
9-Hole Peg Test	r : 25.42 sec l : 2min 47 sec	r: 20.84 sec l: 1min 48 sec
Handgrip force	r: 38/39/31 kg l: 10/9/9 kg	r: 38/40/40 kg l: 11/10/10 kg
Functional Hand Scale (1-5)	r:5 l:3	r:5 l:4
ARAT Score	38/57 pts.	49/57 pts.
AROM left shoulder	ABD – 90°	ABD – 135°

## Results

This is the first-ever reported case of a patient with a chronic ischemic stroke who, although there was virtually no gain of motor function seen during the early phase of recovery and rehabilitation, showed profound improvements in functional motor recovery by using a triple therapy including Cerebrolysin.

We hypothesize that the combination of Cerebrolysin and atDCS induces a milieu of heightened neuroplasticity, possibly by promoting BDNF-mediated synaptic plasticity. We cannot rule out that the observed improvements may primarily present changes in the level of compensation versus real repair and consolidation on the impairment level. Clinically relevant changes were seen in the ARAT score but for example, not in handgrip force. On the other hand, fine movements, speed, and dexterity showed a clinically meaningful improvement in the 9 Hole Peg Test (>20%).

## Discussion

Further studies are needed to confirm our findings that this triple therapy regimen with Cerebrolysin® in combination with anodal transcranial direct current stimulation will enhance the therapeutic benefit of a concomitant neurorehabilitation program, which includes a conventional rehabilitation protocol and repetitive, graded and shaped task-oriented training, on motor function recovery.

## Conflict of Interest

The authors confirm that there are no conflicts of interest.

## References

[1] Berretta A, Tzeng YC, Clarkson AN (2014). Post-stroke recovery: the role of activity-dependent release of brain-derived neurotrophic factor. *Expert Review of Neurotherapeutics*.

[2] Bornstein NM, Guekht A, Vester J, Heiss WD, Gusev E, Hömberg V, Rahlfs VW, Bajenaru O, Popescu BO, Muresanu D (2018). Safety and efficacy of Cerebrolysin in early post-stroke recovery: a meta-analysis of nine randomized clinical trials. *Neurol Sci*.

[3] Alvarez AX, Alvarez I, Iglesias O, Crespo I, Figueroa J, Aleixandre M, Linares C, Granizo E, Garcia-Fantini M, Marey J, Masliah E, Winter S, Muresanu DF, Moessler H (2016). Synergistic Increase of Serum BDNF in Alzheimer Patients Treated with Cerebrolysin and Donepezil: Association with Cognitive Improvement in ApoE4 Cases. *International Journal of Neuropsychopharmacology*.

[4] Fritsch B, Reis J, Martinowich K, Schambra HM, Ji Y, Cohen LG, Fritsch Lu B. B. (2010). Direct current stimulation promotes BDNF-dependent synaptic plasticity: potential implications for motor learning. *Neuron*.

